# Serum Mammalian Target of Rapamycin (mTOR) Levels in Patients with Post-COVID-19 Fibrotic-like Lung Changes: A Cross-Sectional Study

**DOI:** 10.3390/diagnostics16060958

**Published:** 2026-03-23

**Authors:** Mert Gebes, Ozge Oral Tapan, Tuba Edgunlu, Emrah Dogan

**Affiliations:** 1 Faculty of Medicine, Mugla Sitki Kocman University, Mugla 48000, Türkiye; mertg5f67@gmail.com; 2 Faculty of Medicine, Department of Pulmonology, Mugla Sitki Kocman University, Mugla 48000, Türkiye; 3 Faculty of Medicine, Department of Medical Biology, Mugla Sitki Kocman University, Mugla 48000, Türkiye; tedgunlu@gmail.com; 4 Faculty of Medicine, Department of Radiology, Mugla Sitki Kocman University, Mugla 48000, Türkiye; dr_e_dogan@hotmail.com

**Keywords:** post-COVID-19 fibrotic-like lung changes, mTOR, COVID-19, pulmonary sequelae

## Abstract

**Background/Objectives:** Post-COVID-19 fibrotic-like lung changes (PC19-FLC), which may represent persistent post-inflammatory abnormalities or early fibrotic remodeling, have emerged as an important long-term pulmonary sequela following SARS-CoV-2 infection. However, the underlying pathogenic mechanisms remain incompletely understood. This study aimed to investigate the potential association between mammalian target of rapamycin (mTOR) activity and the presence of PC19-FLC. **Methods:** This single-center, cross-sectional study included 70 patients who met the predefined inclusion criteria. Participants were categorized according to the presence or absence of PC19-FLC on chest computed tomography. Demographic, laboratory, and radiological data were collected. Serum mTOR levels were measured using enzyme-linked immunosorbent assay (ELISA). **Results:** Serum mTOR levels and modified Medical Research Council (mMRC) dyspnea scores were significantly higher in patients with PC19-FLC compared with those without fibrotic-like changes. Receiver operating characteristic (ROC) curve analysis identified a serum mTOR cut-off value of 6.15 ng/mL (sensitivity 83%, specificity 94%) for discriminating patients with PC19-FLC in this cohort. Serum mTOR levels were significantly correlated with forced vital capacity (FVC%), mMRC dyspnea score, and peripheral oxygen saturation (SpO_2_). **Conclusions:** Increased serum mTOR levels were associated with the presence of fibrotic-like lung changes after COVID-19 and may help distinguish patients with such CT abnormalities in this cohort. Higher mTOR levels were also associated with greater dyspnea severity, lower lung volumes, and reduced peripheral oxygen saturation. These findings suggest a potential role of mTOR signaling in post-COVID-19 pulmonary sequelae and warrant further investigation in larger, multicenter studies.

## 1. Introduction

Since March 2020, millions of people worldwide have been infected with severe acute respiratory syndrome coronavirus 2 (SARS-CoV-2). Although mortality associated with coronavirus disease 2019 (COVID-19) has declined, long-term health sequelae remain a significant clinical challenge. The period between 4 and 12 weeks following acute infection is defined as ongoing symptomatic COVID-19 [1]. Long COVID-19 refers to a heterogeneous syndrome characterized by persistent or newly emerging symptoms lasting more than three months after the onset of COVID-19 that cannot be explained by alternative diagnoses [2].

Multiple viral- and immune-mediated mechanisms of lung injury in COVID-19 have been described [3]. In survivors of severe disease, iatrogenic factors such as oxygen toxicity and ventilator-associated barotrauma may also contribute to pulmonary injury [4]. Post-COVID-19 pulmonary fibrosis (PC19-PF) may arise as a consequence of chronic inflammation or may reflect an idiopathic, genetic, or age-related fibroproliferative process [5]. During the inflammatory phase of acute respiratory distress syndrome (ARDS), cytokine storm as well as epithelial and endothelial injury may initiate and promote fibrotic remodeling of lung tissue [6]. A prolonged inflammatory response can further damage the respiratory epithelium and vascular endothelium, resulting in cytokine-mediated tissue injury. SARS-CoV-2 may directly contribute to pulmonary fibrosis through its immunological effects on alveolar epithelial cells. By binding to the angiotensin-converting enzyme 2 (ACE2) receptor, the virus may activate profibrotic signaling cascades, particularly in individuals with genetic susceptibility and/or subclinical interstitial lung disease (ILD) [7].

The phosphoinositide 3-kinase (PI3K)/protein kinase B (AKT)/mammalian target of rapamycin (mTOR) signaling pathway is a central regulator of cellular metabolism, proliferation, differentiation, and survival [8]. Dysregulation of this pathway has been implicated in a variety of pathological conditions, including malignancies, immune-mediated diseases, and fibroproliferative disorders such as pulmonary fibrosis. The mTOR complex is a serine/threonine kinase that functions as a key regulator of the balance between anabolic and catabolic processes and is often described as the master regulator of this pathway.

The aim of this study was to investigate the association between serum mTOR levels and fibrotic-like lung abnormalities on CT in patients after COVID-19 infection.

## 2. Materials and Methods

This single-center, cross-sectional study was conducted after approval was obtained from the Clinical Research Ethics Committee of Muğla Sıtkı Koçman University Faculty of Medicine (24 August 2022; approval no. 14/XIV). Patients admitted to the adult pulmonology outpatient clinic were evaluated, and those who had recovered from COVID-19 pneumonia were enrolled between October 2022 and March 2023. Patients who met the inclusion criteria, agreed to participate, and provided written informed consent were included in the study.

### 2.1. Patient Selection and Study Design

**Figure 1:**
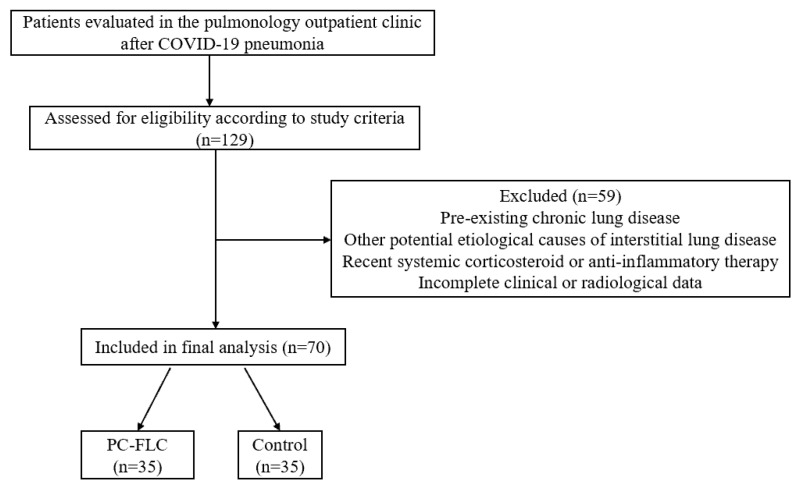
Study flow diagram of patient selection and group allocation. PC-FLC: Post-COVID fibrotic-like lung changes; Control: No fibrotic CT abnormalities.

The main inclusion criterion was a minimum of 12 weeks having elapsed since acute SARS-CoV-2 infection. Patients were excluded if they had a history of pre-existing chronic lung disease, other potential etiological causes of interstitial lung disease, recent use of systemic corticosteroids or other anti-inflammatory medications, or incomplete clinical or radiological data that could affect the reliability of the analysis. Patients without sequelae on thoracic computed tomography (CT) were assigned to the control group. Patients whose thoracic CT scans demonstrated features consistent with septal thickening, traction bronchiectasis, honeycombing, reticulation, or parenchymal bands were reviewed. After exclusion of other potential etiological causes of interstitial lung disease, these patients were classified as the post-COVID fibrotic-like lung changes (PC-FLC) group (Figure 1).

Demographic characteristics, smoking history, comorbidities, spirometry results, peripheral oxygen saturation values, and blood samples were collected from all participants. Comorbidities were assessed using the Charlson Comorbidity Index (CCI). Blood samples were centrifuged, and serum specimens were stored at −80 °C until analysis. Modified Medical Research Council (mMRC) dyspnea scores, spirometric measurements, clinical data, and thoracic CT findings were re-evaluated for all patients.

### 2.2. Laboratory Analysis

Serum mTOR levels were measured using a commercially available ELISA kit (BT Lab, Bioassay Technology Laboratory, Shanghai, China; Cat. No: E3693HU) following the manufacturer’s protocol. Prior to analysis, serum samples were stored at −80 °C to ensure protein stability. All reagents and samples were brought to room temperature before use. To ensure reproducibility, all standards and serum samples were analyzed in duplicate, and the mean optical density (OD) values were used for final concentration calculations. Optical density was measured at 450 nm using a SpectraMax i3 microplate reader (Molecular Devices, San Jose, CA, USA). The standard curve was generated using serial dilutions (24, 12, 6, 3, and 1.5 ng/mL). The intra-assay coefficient of variation (CV) was maintained below 10%, ensuring the technical consistency of the measurements. Laboratory personnel were blinded to the clinical and radiological status of the study participants.

### 2.3. Radiological Evaluation

Thoracic computed tomography (CT) scans were retrieved retrospectively from the radiology department’s digital archive. Imaging had been performed using 256-slice Toshiba TCT-60 AX and 4-slice Siemens Somatom scanners (tube voltage: 120 kV; tube current modulation: 100–250 mAs; spiral pitch factor: 0.98; collimation width: 0.625 mm). CT images were evaluated on a high-resolution diagnostic workstation by an experienced thoracic radiologist who was blinded to the clinical and laboratory data. The images were reviewed by a single radiologist, and interobserver agreement could therefore not be assessed.

Fibrotic-like changes were defined as the presence of reticulation, traction bronchiectasis, interlobular septal thickening, parenchymal bands, or honeycombing. However, in the post-COVID setting, these findings may represent persistent post-inflammatory or residual lung abnormalities rather than established irreversible fibrosis.

### 2.4. Statistical Analysis

Sample size calculation was based on an expected area under the receiver operating characteristic (ROC) curve (AUC) of 0.70 for serum mTOR levels. The expected AUC value of 0.70 was selected based on conservative assumptions from previous biomarker studies in pulmonary diseases. To achieve statistical significance with an alpha level of 0.05 and a power of 80% (β = 0.20), a minimum of 30 patients per group was required. Considering potential dropouts, a total of 70 patients were included.

Statistical analyses were performed using SPSS version 22.0 (IBM Corp., Armonk, NY, USA). Continuous variables were assessed for normality using the Kolmogorov–Smirnov test. Normally distributed variables were expressed as mean ± standard deviation (SD), whereas non-normally distributed variables were presented as median with interquartile range (IQR). Categorical variables were expressed as frequencies and percentages. Comparisons between groups were performed using Student’s *t*-test for normally distributed variables and the Mann–Whitney U test for non-normally distributed variables. The chi-square test was used for comparisons of categorical variables. Correlations between serum mTOR levels and clinical parameters were evaluated using Spearman’s rank correlation coefficient due to the non-normal distribution of biomarker values. Multivariable logistic regression analysis was performed to evaluate the association between serum mTOR levels and fibrotic-like CT abnormalities after adjustment for potential confounders, including age, sex, smoking status, and indicators of disease severity. Effect sizes were reported as odds ratios (ORs) with 95% confidence intervals (CIs). Receiver operating characteristic (ROC) curve analysis was used to assess the discriminative ability of serum mTOR levels for fibrotic-like CT abnormalities. The optimal cut-off value was determined using the Youden index. A two-sided *p* value < 0.05 was considered statistically significant.

## 3. Results

The mean age of patients with PC19-FLC (*n* = 35) was significantly higher than that of the control group without fibrotic-like lung changes on thoracic CT. Sex distribution and smoking status were similar between the two groups. Comorbidities, hospitalization during acute COVID-19, and mechanical ventilation were more frequent in the PC19-FLC group (Table 1).

Spirometric parameters (FVC% and FEV1%) and peripheral oxygen saturation (SpO_2_) were significantly reduced in patients with PC19-FLC, whereas serum mTOR levels and mMRC dyspnea scores were significantly higher than in the control group (Table 2).

Among patients with PC19-FLC, reticulation (38.6%) was the most frequently observed radiological pattern, followed by septal thickening (22.9%), parenchymal bands (21.4%), bronchiectasis (12.9%), and honeycombing (4.2%).

**Figure 2:**
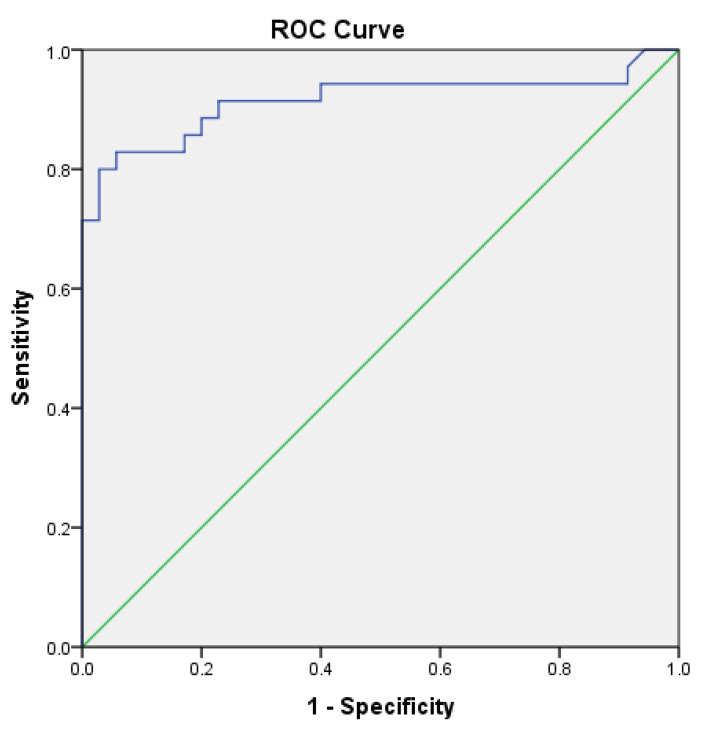
ROC curve of serum mTOR levels for discriminating patients with post-COVID fibrotic-like lung changes.

Receiver operating characteristic (ROC) curve analysis demonstrated that serum mTOR levels could discriminate patients with post-COVID fibrotic-like lung changes (PC19-FLC) from controls. The area under the curve (AUC) was 0.915 (95% CI 0.838–0.991; *p* < 0.001). The optimal cut-off value was 6.15 ng/mL, corresponding to a sensitivity of 83% and a specificity of 94% (Figure 2).

Since the variables included in the correlation analyses did not demonstrate normal distribution, Spearman rank correlation analysis was used. The correlation analysis revealed significant associations between MTOR levels and several clinical and respiratory parameters.

Clinical Outcomes and Comorbidities: A moderate and statistically significant positive correlation was found between MTOR levels and the duration of hospitalization (rho = 0.509, *p* < 0.001). MTOR levels also showed significant positive correlations with the Charlson Comorbidity Index (CCI) (rho = 0.367, *p* = 0.002), patient age (rho = 0.253, *p* = 0.035), and the duration of mechanical ventilation (MV) (rho = 0.294, *p* = 0.014).

Respiratory Function and Dyspnea: Regarding respiratory parameters, mTOR levels exhibited a significant negative correlation with SpO_2_ (rho = −0.502, *p* < 0.001), FVC (%) (rho = −0.430, *p* < 0.001) and FEV1 (%) (rho = −0.261, *p* = 0.029). These findings suggest that higher mTOR levels are associated with reduced oxygen saturation and impaired forced vital capacity. Furthermore, a significant positive correlation was identified between mTOR levels, and mMRC dyspnea scores (rho = 0.380, *p* < 0.001), indicating that patients with higher mTOR levels reported greater levels of breathlessness.

Multivariable logistic regression analysis was performed to identify factors independently associated with fibrotic-like lung changes on CT. The model included age, Charlson Comorbidity Index, duration of hospitalization, duration of mechanical ventilation, and serum mTOR levels.

In the adjusted model, serum mTOR level was the only variable independently associated with fibrotic-like CT abnormalities. Higher serum mTOR levels were significantly associated with an increased likelihood of fibrotic-like lung changes (OR = 3.91, 95% CI: 1.84–8.34, *p* < 0.001).

None of the other variables included in the model, including age, comorbidity burden, or duration of mechanical ventilation, showed a statistically significant association with fibrotic-like CT abnormalities. Hospitalization duration demonstrated a positive trend but did not reach statistical significance (OR = 1.27, *p* = 0.065) (Table 3).

## 4. Discussion

Our findings suggest that increased circulating mTOR levels may be associated with fibrotic-like CT abnormalities and adverse clinical parameters, including reduced FVC, lower oxygen saturation, and higher dyspnea scores, supporting a potential link between mTOR pathway activity and the severity of post-COVID pulmonary involvement. Although the present findings do not establish causality, they suggest that circulating mTOR levels may reflect ongoing pathophysiological processes related to post-COVID lung injury and remodeling. Furthermore, serum mTOR levels demonstrated good discriminatory ability for identifying patients with fibrotic-like CT abnormalities in this cohort (AUC = 0.915, sensitivity 83%, specificity 94%). Higher serum mTOR levels also remained independently associated with post-COVID fibrotic-like lung changes (OR = 3.91, 95% CI: 1.84–8.34, *p* < 0.001). The proposed cut-off value of 6.15 ng/mL should be interpreted in the context of supportive clinical assessment rather than as a stand-alone diagnostic threshold. In clinical practice, this level may help identify patients who could benefit from closer radiological or functional follow-up, particularly in individuals with persistent respiratory symptoms after COVID-19. Therefore, the threshold may be better interpreted as a supportive risk-stratification marker rather than a rule-in or rule-out diagnostic test. However, these findings should be interpreted cautiously, as the results are derived from a single-center cross-sectional dataset and require validation in larger independent cohorts.

Approximately 40% of patients with severe COVID-19 pneumonia develop acute respiratory distress syndrome (ARDS) [9]. Extensive alveolar damage and loss of type II pneumocytes during severe ARDS lead to hyaline membrane formation, alveolar thickening, and basement membrane disruption, ultimately promoting fibrotic remodeling of lung tissue. Previous studies have highlighted the duration of ARDS as a key determinant of PC19-PF development [10]. Consistent with the literature, patients with PC19-FLC in our study had longer ICU stays and longer durations of mechanical ventilation. Moreover, mechanical ventilation and length of hospitalization were significantly associated with fibrotic lung changes.

Another proposed mechanism of postviral pulmonary fibrosis involves virus-induced upregulation of profibrotic cytokines, such as transforming growth factor-β1 (TGF-β1), along with increased oxidative stress [11]. In genetically predisposed individuals, repeated microinjuries to the alveolar epithelium and basement membrane trigger persistent activation of epithelial and immune cells, leading to the secretion of proinflammatory cytokines and chemokines including tumor necrosis factor-α (TNF-α), interleukin-1 (IL-1), and monocyte chemoattractant protein-1 (MCP-1) [12]. Alterations in mTOR signaling are closely linked to dysregulated autophagy, chronic inflammation, and abnormal cell growth and survival, all of which contribute to fibrogenesis [8]. Experimental studies have shown that mTOR inhibitors may reduce type II pneumocyte injury and attenuate fibrotic responses [13]. Additionally, inflammatory mediators such as TGF-β, vascular endothelial growth factor (VEGF), interleukin-6 (IL-6), and TNF-α play crucial roles in the initiation and progression of pulmonary fibrosis [14,15]. The mTOR complex indirectly promotes fibrosis by enhancing TGF-β signaling [16,17]. In line with these mechanisms, increased mTOR activity may therefore contribute to persistent inflammatory signaling and aberrant tissue repair processes that promote fibrotic remodeling following viral lung injury in our patients. Acute severity is a known risk factor for post-COVID pulmonary sequelae and may represent an important source of confounding in biomarker studies. Although multivariable adjustment was performed in our study results, residual confounding related to disease severity cannot be fully excluded.

Chun et al. [18] investigated several fibrotic biomarkers, including matrix metalloproteinase-7, hepatocyte growth factor, and lipocalin-2, in patients with COVID-19 and demonstrated positive correlations with ICU admission and post-recovery pulmonary function tests. Restrictive ventilatory impairment in pulmonary fibrosis is characterized by reductions in total lung capacity (TLC), functional residual capacity (FRC), and residual volume (RV) [19]. FVC is a well-established surrogate endpoint in clinical trials of idiopathic pulmonary fibrosis (IPF) [20]. In our study, FVC% was reduced in patients with PC19-FLC and showed a significant negative correlation with serum mTOR levels. Although diffusion capacity for carbon monoxide (DLCO) and exercise testing more accurately reflect fibrotic severity, FVC remains a practical and widely used parameter for disease monitoring. However, a limitation of our study is the absence of DLCO and exercise test data, which might have provided additional insight into functional impairment.

Dyspnea is the most commonly reported persistent symptom following COVID-19 infection [21]. In our cohort, patients with PC19-FLC had higher mMRC dyspnea scores, which showed a positive correlation with serum mTOR levels, suggesting that mTOR activity may reflect clinical symptom burden.

Peripheral oxygen saturation measured by pulse oximetry is a simple, noninvasive method that has prognostic value in IPF [22]. In this study, a negative correlation was observed between serum mTOR levels and SpO_2_, indicating that increased mTOR activity may be associated with impaired oxygenation and more advanced fibrotic involvement.

Patients with PC19-FLC were older and had a higher burden of comorbidities compared with those without PC19-FLC. However, the observed age difference between groups should be considered a potential limitation, as aging itself may influence mTOR pathway activity. Smoking history was similar between the groups. Previous studies have identified older age, comorbidities, smoking, and chronic alcohol use as risk factors for PC19-PF [23,24]. Consistent with these findings, Charlson Comorbidity Index (CCI) scores were higher in the PC19-FLC group in our cohort. In addition, genetic and epigenetic susceptibility may also contribute to the development of fibrotic lung changes [25].

Although the highest incidence of long COVID was reported during the early waves of the pandemic, persistent sequelae continue to be observed with emerging variants [26]. Several studies suggest a lower risk of long COVID following Omicron infection compared with Delta and earlier variants [27]. Nevertheless, all patients in our cohort were infected during the Omicron wave, indicating that PC19-FLC may still develop even with newer variants.

Radiological abnormalities persist in a substantial proportion of patients following COVID-19 infection. Previous studies reported residual CT findings in 56.7% of patients at one year [28], and up to 81% of severely ill patients at 12 months [29]. Interstitial thickening, irregular interfaces, reticular patterns, and parenchymal bands have been proposed as predictors of fibrosis in survivors of severe COVID-19 pneumonia [30]. In our cohort, reticulation, septal thickening, and parenchymal bands were the most common radiological patterns, whereas honeycombing—typical of IPF—was rare, suggesting a fibrotic phenotype distinct from classic IPF [31].

Emerging evidence indicates that chemokines such as CXCL9, CXCL10, and CCL5, as well as gene expression differences involved in immune cell migration and fibrogenesis, are altered in both IPF and COVID-19 [32]. However, the clinical course and radiological patterns of post-COVID fibrotic lung disease differ from those of IPF. Early identification of patients at risk for developing pulmonary fibrosis after COVID-19 is therefore critical for timely intervention. Currently, no reliable clinical or laboratory biomarkers exist for the early diagnosis, prognostic assessment, or monitoring of PC19-PF. To our knowledge, this is the first study to evaluate serum mTOR levels using ELISA as a potential indicator of patients with fibrotic-like CT abnormalities in post-COVID patients.

This study has several limitations that should be considered when interpreting the findings. First, the sample size was relatively small and the study was conducted at a single center, which may limit statistical precision, model stability, and the generalizability of the results. Second, physiologic characterization of lung disease was limited because diffusion capacity for carbon monoxide (DLCO) and exercise capacity measurements were not available. The absence of these parameters may have resulted in incomplete phenotyping and potential misclassification of disease severity. Third, baseline differences between groups, including age, comorbidity burden, and indicators of acute disease severity such as hospitalization and ICU admission, may represent residual confounding despite multivariable adjustment. In particular, differences in acute COVID-19 severity may partly explain the observed biomarker differences. Fourth, radiological classification was based on CT findings assessed by a single radiologist, and interobserver agreement could not be evaluated. Additionally, some CT findings classified as fibrotic-like changes may represent persistent post-inflammatory abnormalities rather than established fibrosis. Fifth, the diagnostic performance of mTOR and the proposed cut-off value were derived from the same dataset without internal or external validation, which may result in optimistic estimates of diagnostic accuracy. Finally, variability in the time since infection within the ≥12-week inclusion window may have also influenced both CT findings and biomarker levels. In addition, patient enrollment occurred during a specific late-pandemic period, reflecting the circulating variant and clinical care context at that time. Therefore, the applicability of our findings to other pandemic waves, vaccination backgrounds, or treatment eras may be limited. Larger multicenter studies with severity-matched cohorts and validation analyses are required to confirm these findings.

## 5. Conclusions

Elevated serum mTOR levels were associated with fibrotic-like lung changes and adverse respiratory parameters, including reduced FVC, lower oxygen saturation, and higher dyspnea scores in patients with post-COVID lung involvement. Serum mTOR also demonstrated promising diagnostic performance for identifying patients with fibrotic-like CT abnormalities. These findings suggest that circulating mTOR may serve as a potential biomarker reflecting ongoing pathological processes related to post-COVID lung injury and remodeling. However, the proposed mTOR cut-off value should be interpreted with caution. In clinical practice, mTOR may be considered a supportive biomarker that could help identify patients who may benefit from closer radiological and functional follow-up rather than serving as a stand-alone diagnostic test. Given the limitations of this single-center cross-sectional study, including the relatively small sample size and lack of external validation, larger multicenter studies are needed to confirm the diagnostic value and clinical utility of mTOR in post-COVID pulmonary involvement.

## Figures and Tables

**Table 1:** Baseline demographic and clinical characteristics of the study population.

	PC19-FLC (*n* = 35)	Control (*n* = 35)	*p* Value
Age, mean ± SD	64 ± 12	56 ± 15	0.023 *
Sex, *n* (%)			0.621 ᵞ
Male	21 (60)	23 (65)	
Smoking status, *n* (%)			
Current smoker	14 (40)	14 (40)	0.946 ᵞ
Former smoker	7 (20)	6 (17)	
Never smoker	14 (40)	15 (43)	
CCI, median (IQR)	2 (0–6)	1 (0–6)	0.004 ᶲ
Hospitalization during acute COVID-19, *n* (%)	23 (66)	5 (14)	<0.001 ᵞ
ICU admission, *n* (%)	11 (31)	4 (11)	0.041 ᵞ
Length of hospital stay, days, median (IQR)	8 (0–42)	0 (0–18)	<0.001 ᶲ
Duration of mechanical ventilation, days, median (IQR)	0 (0–21)	0 (0–7)	0.023 ᶲ

* Student’s *t*-test; ᵞ Chi-square test; ᶲ Mann–Whitney U test; CCI: Charlson Comorbidity Index; PC19-FLC: Post-COVID fibrotic-like lung changes; Control: No fibrotic CT abnormalities; ICU: Intensive care unit.

**Table 2:** Spirometric measurements, clinical findings, and serum mTOR levels in patients.

	PC19-FLC (*n* = 35)	Control (*n* = 35)	*p* Value
Serum mTOR, median (IQR)	7.35 (6.50–9.26)	4.92 (4.39–5.48)	<0.001 ᶲ
FVC, % predicted (mean ± SD)	82 ± 15	94 ± 14	0.001 *
FEV1, % predicted (mean ± SD)	77 ± 18	87 ± 13	0.011 *
SpO_2_ (%), median (IQR)	95 (93–96)	98 (96–98)	<0.001 ᶲ
mMRC dyspnea scale, median (IQR)	1 (0–2)	1 (0–1)	0.013 ᶲ

* Student’s *t*-test; ᶲ Mann–Whitney U test; PC19-FLC: Post-COVID fibrotic-like lung changes; Control: No fibrotic CT abnormalities; FVC: Forced vital capacity; FEV1: Forced expiratory volume in 1 s; SpO_2_: Peripheral oxygen saturation; mMRC: Modified Medical Research Council.

**Table 3:** Multivariable logistic regression analysis of factors associated with post-COVID fibrotic-like lung changes.

Variable	OR	95% CI	*p* Value
Age	1.02	0.88–1.18	0.793
CCI	0.80	0.20–3.17	0.751
Duration of mechanical ventilation	0.77	0.49–1.20	0.247
Hospital length of stay	1.27	0.99–1.63	0.065
mTOR	3.91	1.84–8.34	<0.001

CCI: Charlson Comorbidity Index; OR: Odds ratio; CI: Confidence interval.

## Data Availability

The data supporting the findings of this study are available from the corresponding author upon reasonable request.
